# Retraction Note: Fluoride exposure and intelligence in school-age children: evidence from different windows of exposure susceptibility

**DOI:** 10.1186/s12889-022-14470-5

**Published:** 2022-11-08

**Authors:** Kaihong Xu, Ning An, Hui Huang, Leizhen Duan, Jun Ma, Jizhe Ding, Tongkun He, Jingyuan Zhu, Zhiyuan Li, Xuemin Cheng, Guoyu Zhou, Yue Ba

**Affiliations:** 1 Department of Environmental Health, School of Public Health, Zhengzhou University, Zhengzhou, 450001 Henan China; 2 Environment and Health Innovation Team, School of Public Health, Zhengzhou University, Zhengzhou, 450001 Henan China; 3 Department of Medical Services, Zhengzhou Central Hospital Affiliated to Zhengzhou University, Zhengzhou, 450001 Henan China; 4 Department of Endemic Disease, Kaifeng Center for Disease Control and Prevention, Kaifeng, 475000 Henan China; 5 The Medical Section, The Eighth People Hospital of Zhengzhou, Zhengzhou, 450000 Henan China; 6 Yellow River Institute for Ecological Protection & Regional Coordinated Development, Zhengzhou University, Zhengzhou, 450001 Henan China

**Retraction Note: BMC Public Health 20, 1657 (2020)**

**https://doi.org/10.1186/s12889-020-09765-4**

The Editor has retracted this article. After publication, concerns were raised regarding the data analysis and conclusions in the paper. The authors have provided raw data, and post-publication review found inconsistencies in methodology and major misinterpretation of the primary result. None of the authors agree to this retraction.

